# The fulcrum: a novel technique for reduction of shoulder dislocations

**DOI:** 10.1007/s43678-025-00907-4

**Published:** 2025-04-15

**Authors:** Paul Carr, Jesse Maracle

**Affiliations:** 1 Northumberland Hills Hospital, Cobourg, ON Canada; 2 Lakeridge Health, Bowmanville, ON Canada

**Keywords:** Emergency medicine, Orthopedic procedures, Shoulder dislocation, Glenohumeral dislocation, Médecine d’urgence, Procédures orthopédiques, Luxation de l’épaule, Luxation glénohumérale

## Introduction

Shoulder dislocation is a common presentation to the emergency department (ED) with recent data from the United States putting the annual incidence at 23.96 per 100,000 persons [1]. In the literature, failure rates are often reported at approximately 8% with delay to treatment being associated with higher failure rates for closed reduction [2]. Many techniques have been described to achieve anatomic reduction of these injuries (Table 1), each with benefits and drawbacks. Factors including patient anatomy, habitus, and type of dislocation can provide challenges achieving reduction. Additionally, bed availability and staff requirements for sedated reductions can delay treatment for patients and impair department flow in general.

**Table 1:** Commonly cited reduction techniques [3]

Davos (Boss-Holzach-Matter) self-reduction technique
External rotation (Hennepin technique) with abduction (Milch technique) if needed
FARES technique
Scapular manipulation
Stimson technique
Traction-countertraction

This case series involves the index case in which a new technique was established and subsequent cases where it was employed to good effect.

## Case 1

A healthy 34-year-old female fell from a step ladder while painting her home. No significant head injury or other acute complication was identified. There was a visible step deformity at the glenohumeral joint and plain radiographs confirmed an anterior dislocation with associated greater tuberosity fracture. Past medical history was otherwise non-contributory.

**Fig. 1:**
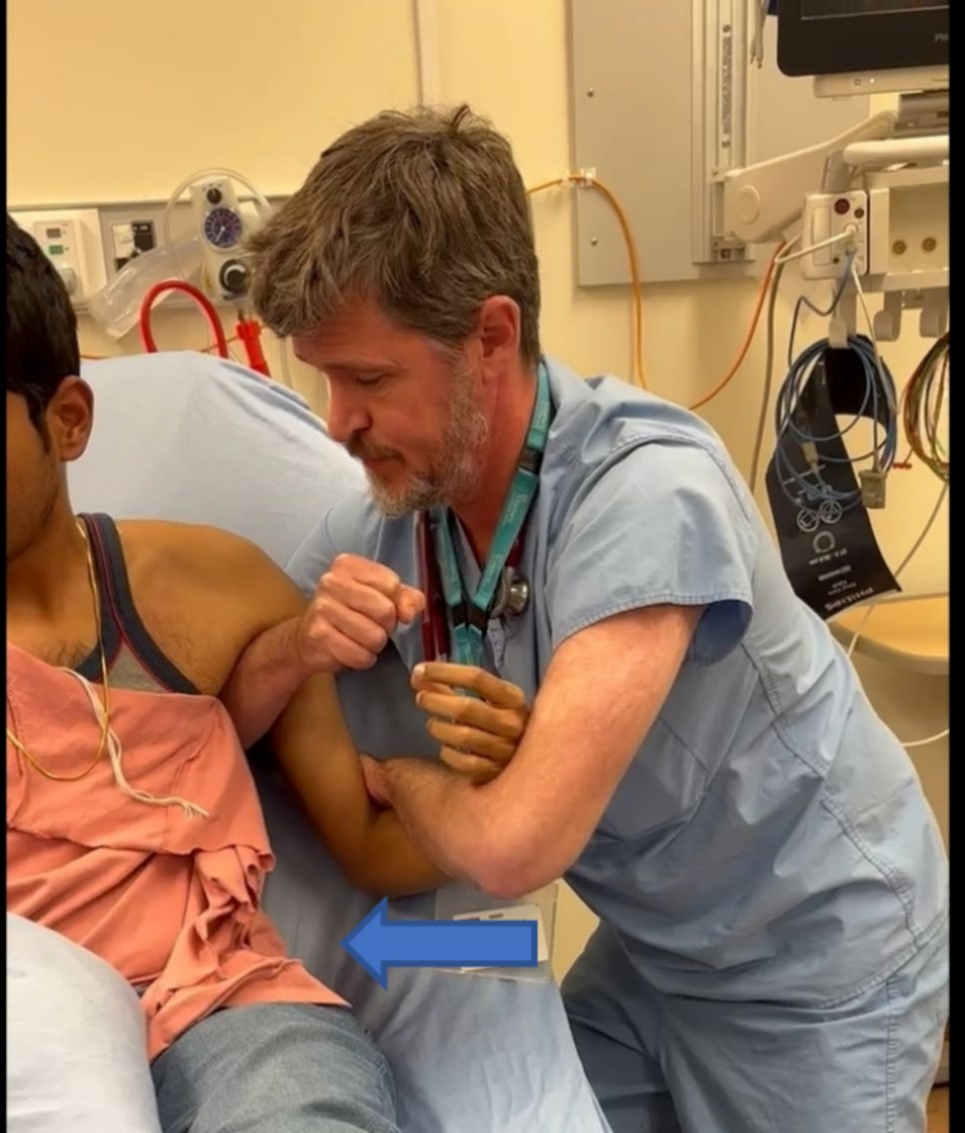
Interlocking forearm grip enables physician to apply force in all axes while the free arm is placed in axilla as the fulcrum point

The patient was given intramuscular ketorolac at triage but there were no beds available in which it would be safe to give significant opioid analgesia. The author approached the patient and offered reduction in the waiting room while seated in her chair. Using a linked forearm grip, gentle traction was applied but the patient struggled to remain upright to allow for sufficient reduction force. The physician placed his forearm into the axilla which allowed him to now apply adduction force to the lateral aspect of the elbow rather than axial traction (Fig. 1, video). The resultant gentle pressure against the lateral aspect of the elbow created a fulcrum which levered the humeral head laterally while helping the scapula to glide medially resulting in a rapid and relatively painless reduction with minimal gross movement of the affected extremity. The patient reported that the procedure added only nominal discomfort to the baseline pain of her injury.

Post reduction x-rays confirmed satisfactory reduction of the humeral head and no exacerbation of the associated fracture. Orthopedic surgery was consulted and the patient was immobilized in a shoulder binder. Follow-up at the fracture clinic was uneventful with no sequelae and the greater tuberosity fracture was managed non-operatively.

## Case 2

A 29-year-old male with very muscular habitus presented with recurrent anterior dislocation. In this case, reduction in the waiting room was not tolerated. A relatively inexperienced physician working in the ED was taught The Fulcrum technique. Initially she applied exclusively axial traction but with a little coaching added adducting force at the elbow with her palm, while levering with the free arm in the axilla, achieving reduction. The physician found the technique easy to learn and required little brute strength.

## Case 3

A 55 year-old 125 kg male was seen at a follow-up appointment with orthopedic surgery for persistent anterior shoulder dislocation and associated glenohumeral and tuberosity fractures 3 days after initial attempt in the ED. Orthopedic surgery brought the patient to the ED for sedation and another trial of closed reduction, where the orthopedic team trialed numerous techniques without success. The patient had challenging habitus and was quite diaphoretic at the time making a satisfactory grip quite challenging. An emergency medicine physician familiar with The Fulcrum technique was performing procedural sedation for the case. He was invited to attempt this technique prior to proceeding to operative reduction. After ensuring safe handover of the sedation, he applied The Fulcrum and an appreciable clunk was felt. The post-reduction x-ray showed satisfactory reduction, and the patient was subsequently managed non-operatively.

## Discussion

The above cases reflect three common clinical scenarios that can lead to poor outcomes involving shoulder dislocation: delay to reduction, the inexperienced practitioner, and the difficult to reduce injury. In all three cases, this novel approach proved easy to execute, well tolerated, and effective. In our practice, the technique has additionally been used successfully for inferior and posterior dislocations with equal degrees of success by adjusting the direction of applied forces.

Anatomically there is the potential for injury to the axillary nerve using this technique. It is important that any lifting (cephalad) force be applied at the elbow and not using the arm in the axilla so as not to interpose neurovascular structures into the joint during reduction.

This case series does not include long term follow-up. Randomized control trials comparing reduction techniques, including long term follow-up, are necessary to demonstrate the comparative effectiveness and safety of this technique, however early results in our practice have been very encouraging.

## Conclusion

The fundamental concept of The Fulcrum technique is that manipulation of a deformity should be focused on the point of injury so that forces are maximal at the area of interest, thereby minimizing total force required and movement of the injured extremity. The technique is usually well tolerated and can be used as the primary approach with or without sedation. Fundamentals of the Fulcrum technique have shown promise in our practice for reduction of other injuries including distal femur fractures and metacarpal fractures. Additional research is required to determine ideal and safe application of the concepts described here.

Orthopedic surgery consultation is strongly recommended prior to attempting any reduction technique in complicated dislocations.

## Supplementary Information

Below is the link to the electronic supplementary material.

Supplementary file1 (DOCX 11 KB)
