# Supplementary material for: The fulcrum: a novel technique for reduction of shoulder dislocations
Source: CJEM. 2025 Apr 15;27(6):491–3. doi: 10.1007/s43678-025-00907-4 (PMC12170694; doi:10.1007/s43678-025-00907-4)
Supplement: Supplementary file 1 — Supplementary file1 (DOCX 11 KB) [file 43678_2025_907_MOESM1_ESM.docx]

The Fulcrum – A Novel Technique for Reduction of Shoulder Dislocations
(Supplementary Media)

Link to video demonstrating The Fulcrum

<https://youtu.be/DnFxtlwI_Sc>
